# Supplementary material for: Prevent with Pleasure: A systematic review of HIV public communication campaigns incorporating a pleasure-based approach
Source: PLOS Glob Public Health. 2025 Mar 10;5(3):e0004005. doi: 10.1371/journal.pgph.0004005 (PMC11892838; doi:10.1371/journal.pgph.0004005)
Supplement: S1 Table — (DOCX) [file pgph.0004005.s002.docx]

**S1 Table**  – **Complete Search Strategy**

| **Search Engine** | **Number of Results** |
| --- | --- |
| EMBASE | 8,808 |
| Web of Science Core Collection | 7,454 |
| PsycInfo | 2,976 |
| Total | 19,238 |
| Total (De-Duplicated) | 11,695 |

**World of Science Core Collection Search**

((AB=(“human immunodeficiency virus” OR “HIV” OR “acquired immune deficiency syndrome” OR “AIDS” OR “sex practice*” OR “condom*” OR “PrEP” OR “Pre-exposure Prophylaxis” OR “sexual behaviour*” OR “safe* sex” OR “circumcision”) OR TI=(“human immunodeficiency virus” OR “HIV” OR “acquired immune deficiency syndrome” OR “AIDS” OR “sex practice*” OR “condom*” OR “PrEP” OR “Pre-exposure Prophylaxis” OR “sexual behaviour*” OR “safe* sex” OR “circumcision”)) AND (TI=("mass media" OR "television" OR "radio" OR "cinema" OR "movie*" OR "social media" OR "social network*" OR "publicity campaign" OR "campaign*" OR "public communication" OR "public service announcement" OR "newspaper" OR "magazine" OR "entertainment education" or "social market*" OR "brochure" OR "flyer" OR "educational literature" OR "billboard" OR "Twitter" OR "Facebook" OR "Instagram" OR "YouTube" OR "Snapchat" OR "blog" OR "mobile app*" OR "text messag*" OR "sex* education*" OR "reproducti* education" OR "comprehensive sex* education") OR AB=("mass media" OR "television" OR "radio" OR "cinema" OR "movie*" OR "social media" OR "social network*" OR "publicity campaign" OR "campaign*" OR "public communication" OR "public service announcement" OR "newspaper" OR "magazine" OR "entertainment education" or "social market*" OR "brochure" OR "flyer" OR "educational literature" OR "billboard" OR "Twitter" OR "Facebook" OR "Instagram" OR "YouTube" OR "Snapchat" OR "blog" OR "mobile app*" OR "text messag*" OR "sex* education*" OR "reproducti* education" OR "comprehensive sex* education"))) AND PY=(2010-2023)

**PsychInfo Search**

APA PsycInfo <1806 to February Week 4 2024>

1 ("human immunodeficiency virus" or "HIV" or "acquired immune deficiency syndrome" or "AIDS" or "sex practice*" or "condom*" or "PrEP" or "Pre-exposure Prophylaxis" or "sexual behaviour*" or "safe* sex" or "circumcision").tw. 88072

2 ("mass media" or "television" or "radio" or "cinema" or "movie*" or "social media" or "social network*" or "publicity campaign" or "campaign*" or "public communication" or "public service announcement" or "newspaper" or "magazine" or "entertainment education" or "social market*" or "brochure" or "flyer" or "educational literature" or "billboard" or "Twitter" or "Facebook" or "Instagram" or "YouTube" or "Snapchat" or "blog" or "mobile app*" or "text messag*" or "sex* education*" or "reproducti* education" or "comprehensive sex* education").tw. 137551

3 1 and 2 5326

4 limit 3 to yr="2010 - 2023" 3316

**EMBASE Search**

Embase <1974 to 2024 February 27>

1 ("human immunodeficiency virus" or "HIV" or "acquired immune deficiency syndrome" or "AIDS" or "sex practice*" or "condom*" or "PrEP" or "Pre-exposure Prophylaxis" or "sexual behaviour*" or "safe* sex" or "circumcision").tw. 623278

2 ("mass media" or "television" or "radio" or "cinema" or "movie*" or "social media" or "social network*" or "publicity campaign" or "campaign*" or "public communication" or "public service announcement" or "newspaper" or "magazine" or "entertainment education" or "social market*" or "brochure" or "flyer" or "educational literature" or "billboard" or "Twitter" or "Facebook" or "Instagram" or "YouTube" or "Snapchat" or "blog" or "mobile app*" or "text messag*" or "sex* education*" or "reproducti* education" or "comprehensive sex* education").tw. 282605

3 1 and 2 12065

4 limit 3 to yr="2010 - 2023" 8767
