# Supplementary material for: Prevent with Pleasure: A systematic review of HIV public communication campaigns incorporating a pleasure-based approach
Source: PLOS Glob Public Health. 2025 Mar 10;5(3):e0004005. doi: 10.1371/journal.pgph.0004005 (PMC11892838; doi:10.1371/journal.pgph.0004005)
Supplement: S2 Table — (DOCX) [file pgph.0004005.s003.docx]

**S2 Table**  **– Quality Assessment**

*Randomised Controlled Trials* (Johanna Briggs Institute Critical Appraisal Tool - <https://jbi.global/critical-appraisal-tools>)

|  | Was true randomisation used for assignment of participants to treatment group? | Was allocation to treatment groups concealed? | Were treatment groups similar at baseline? | Were participants blind to treatment assignment? | Were those delivering the treatment blind to treatment assignment? | Were treatment groups treated identically other than the intervention of interest? | Were outcome assessors blind to treatment assignment? | Were outcomes measured in the same way for treatment groups? | Were outcomes measured in a reliable way? | Was follow up complete and if not, were differences between groups in terms of their follow up adequately described and analysed? | Were participants analysed in the groups to which they were randomised? | Was appropriate statistical analysis used? | Was the trial design appropriate and any deviations from the standard RCT design (individual randomisation, parallel groups) accounted for in the conduct and analysis of the trial? | Overall Risk of bias |
| --- | --- | --- | --- | --- | --- | --- | --- | --- | --- | --- | --- | --- | --- | --- |
| Bull, 2012 | Unclear | Unclear | Yes | No | Not Applicable | No | No | Yes | Yes | Yes | Yes | Unclear | Yes | Moderate |
| Schnall, 2022 | Yes | Yes | Yes | Yes | Not Applicable | No | Unclear | Yes | Yes | Yes | Yes | Yes | Yes | Low |
| McCarthy, 2016 | Yes | Yes | No | No | Not Applicable | No | Yes | Yes | Yes | No | Yes | Unclear | Yes | Moderate |
| Free, 2022 | Yes | Yes | Yes | No | Not Applicable | No | Yes | Yes | Yes | Yes | Yes | Yes | Yes | Moderate |
| Jones, 2012 | Yes | Yes | Unclear | No | Not Applicable | No | Unclear | Yes | No | No | Yes | Unclear | Yes | Moderate |
| Leiby, 2016 | Unclear | Unclear | Yes | Unclear | Not Applicable | No | Unclear | Yes | Yes | Yes | Yes | Yes | Yes | Low |
| Patterson, 2020 | Yes | Unclear | No | Unclear | Not Applicable | No | Unclear | Yes | Yes | Yes | Yes | Yes | Yes | Low |
| Reback, 2021 | Yes | Unclear | No | Unclear | Not Applicable | No | Unclear | Yes | Yes | No | Yes | Yes | Yes | Moderate |
| Tan, 2022 | Yes | Unclear | No | No | Not Applicable | No | Unclear | Yes | Yes | No | Yes | Yes | Yes | Moderate |
| Tang, 2016 | Unclear | Yes | Yes | Yes | Not Applicable | No | Yes | Yes | Yes | No | Yes | Unclear | Yes | Low |
| Ybarra, 2021 | Yes | No | Yes | Yes | Not Applicable | No | No | Yes | Yes | Yes | Yes | Unclear | Yes | Moderate |
| Ybarra, 2018 | Yes | No | No | Yes | Not Applicable | No | No | Yes | Yes | Unclear | Yes | Yes | Yes | Moderate |
| Ybarra, 2017 | Yes | No | Yes | Yes | Not Applicable | No | No | Yes | Yes | Yes | Yes | Yes | Yes | Moderate |
| Aladin, 2023 | Unclear | Unclear | No | Unclear | Not Applicable | No | No | Yes | Yes | Unclear | Yes | Unclear | Yes | Moderate |

*Quasi-Experimental Studies (*Johanna Briggs Institute Critical Appraisal Tool - <https://jbi.global/critical-appraisal-tools>)

|  | Is it clear in the study what is the ‘cause’ and what is the ‘effect’ (i.e. there is no confusion about which variable comes first)? | Were the participants included in any comparisons similar? | Were the participants included in any comparisons receiving similar treatment/care, other than the exposure or intervention of interest? | Was there a control group? | Were there multiple measurements of the outcome both pre and post the intervention/exposure? | Was follow up complete and if not, were differences between groups in terms of their follow up adequately described and analysed? | Were the outcomes of participants included in any comparisons measured in the same way? | Were outcomes measured in a reliable way? | Was appropriate statistical analysis used? | Overall risk of bias |
| --- | --- | --- | --- | --- | --- | --- | --- | --- | --- | --- |
| Boudewyns, 2018 | Yes | Not Applicable | Not Applicable | No | Yes | Unclear | Not Applicable | Yes | Yes | Low |
| Jones, 2021 | Yes | Not Applicable | Not Applicable | No | Yes | Unclear | Not Applicable | Yes | Not Applicable | Low |
| Jones, 2015 | Yes | Yes | Unclear | Yes | Yes | Unclear | Not Applicable | Yes | Yes | Low |
| Nambiar, 2011 | Yes | No | Yes | Yes | Yes | Unclear | Yes | Yes | Yes | Low |
| Prati, 2016 | Yes | Not Applicable | Not Applicable | No | Yes | Not Applicable | Not Applicable | Yes | Yes | Low |
| Prati, 2016 | Yes | Yes | Yes | Yes | Yes | Yes | Yes | Yes | Yes | Low |

*Qualitative Research (*Johanna Briggs Institute Critical Appraisal Tool - <https://jbi.global/critical-appraisal-tools>)

|  | Is there congruity between the stated philosophical perspective and the research methodology? | Is there congruity between the research methodology and the research question or objectives? | Is there congruity between the research methodology and the methods used to collect data? | Is there congruity between the research methodology and the representation and analysis of data? | Is there congruity between the research methodology and the interpretation of results? | Is there a statement locating the researcher culturally or theoretically? | Is the influence of the researcher on the research, and vice- versa, addressed? | Are participants, and their voices, adequately represented? | Is the research ethical according to current criteria or, for recent studies, and is there evidence of ethical approval by an appropriate body? | Do the conclusions drawn in the research report flow from the analysis, or interpretation, of the data? | Overall risk of bias |
| --- | --- | --- | --- | --- | --- | --- | --- | --- | --- | --- | --- |
| Keene, 2020 | Yes | Yes | Yes | Yes | Yes | No | No | Yes | Yes | Yes | Moderate |
| Cordoba, 2021 | Yes | Yes | Yes | Yes | Yes | No | No | Yes | Yes | Yes | Moderate |
| Berendes, 2023 | Yes | Yes | Yes | Yes | Yes | Yes | Yes | Yes | Yes | Yes | Low |
| Hightow-Weidman, 2011 | Yes | Yes | Yes | Yes | Yes | No | No | Yes | Yes | Yes | Moderate |
| Nielson, 2020 | Not Applicable | Yes | Yes | Yes | Yes | No | No | Yes | Yes | Yes | Moderate |
| Pedrana, 2014 | Yes | Yes | Yes | Yes | Yes | No | No | Yes | Yes | Yes | Moderate |
| Widman, 2016 | Yes | Yes | Yes | Yes | Yes | No | No | Yes | Unclear | Yes | Moderate |
| Schnall, 2018 | Not Applicable | Yes | Yes | Yes | Yes | No | No | Yes | Yes | Yes | Moderate |

*Pre-Post Study (NIH Study Quality Assessment Tool -* [*https://www.nhlbi.nih.gov/health-topics/study-quality-assessment-tools*](https://www.nhlbi.nih.gov/health-topics/study-quality-assessment-tools)*)*

|  | Was the study question or objective clearly stated? | Were eligibility/selection criteria for the study population prespecified and clearly described? | Were the participants in the study representative of those who would be eligible for the test/service/intervention in the general or clinical population of interest? | Were all eligible participants that met the prespecified entry criteria enrolled? | Was the sample size sufficiently large to provide confidence in the findings? | Was the test/service/intervention clearly described and delivered consistently across the study population? | Were the outcome measures prespecified, clearly defined, valid, reliable, and assessed consistently across all study participants? | Were the people assessing the outcomes blinded to the participants' exposures/interventions? | Was the loss to follow-up after baseline 20% or less? Were those lost to follow-up accounted for in the analysis? | Did the statistical methods examine changes in outcome measures from before to after the intervention? Were statistical tests done that provided p values for the pre-to-post changes? | Were outcome measures of interest taken multiple times before the intervention and multiple times after the intervention (i.e., did they use an interrupted time-series design)? | If the intervention was conducted at a group level (e.g., a whole hospital, a community, etc.) did the statistical analysis take into account the use of individual-level data to determine effects at the group level? | Overall risk of bias |
| --- | --- | --- | --- | --- | --- | --- | --- | --- | --- | --- | --- | --- | --- |
| Ignacio, 2019 | Yes | Yes | Yes | Unclear | No | Yes | Yes | Unclear | No | Not Applicable | No | Not Applicable | Moderate |
| Jones, 2018 | Yes | Yes | Yes | Unclear | Yes | Yes | Yes | Unclear | Unclear | Yes | No | Not Applicable | Low |
| Shrestha, 2020 | Yes | Yes | Yes | Unclear | No | Yes | Yes | Unclear | Unclear | Not Applicable | No | Not Applicable | Low |
| Yao, 2018 | Yes | Yes | Yes | Unclear | Yes | Yes | Yes | Unclear | No | Yes | Yes | Not Applicable | Low |
| Sun, 2020 | Yes | Yes | Yes | Unclear | No | Yes | No | Unclear | Unclear | Yes | No | Not Applicable | Moderate |
| Wilkinson, 2016 | Yes | Yes | Yes | Unclear | Yes | Yes | Yes | Unclear | Yes | Yes | Yes | Not Applicable | Low |

*Analytical Cross-sectional Studies (*[*https://jbi.global/critical-appraisal-tools*](https://jbi.global/critical-appraisal-tools)*)*

|  | Were the criteria for inclusion in the sample clearly defined? | Were the study subjects and the setting described in detail? | Was the exposure measured in a valid and reliable way? | Were objective, standard criteria used for measurement of the condition? | Were confounding factors identified? | Were strategies to deal with confounding factors stated? | Were the outcomes measured in a valid and reliable way? | Was appropriate statistical analysis used? | Overall risk of bias |
| --- | --- | --- | --- | --- | --- | --- | --- | --- | --- |
| Phillips, 2020 | Yes | Yes | Yes | Yes | Yes | Yes | Yes | Yes | Low |
| Phillips, 2020 | Yes | Yes | Yes | Yes | No | No | Yes | Yes | Moderate |
| Dehlin, 2019 | No | Yes | Yes | Yes | No | No | Yes | Yes | Moderate |
| Habarta, 2017 | Yes | Yes | Yes | Yes | Yes | Yes | Yes | Yes | Low |
| Badal, 2019 | Yes | Yes | Yes | Yes | Yes | Yes | Yes | Yes | Low |
| Cao, 2019 | Yes | Yes | Yes | Yes | Yes | Yes | Yes | Yes | Low |
| Flowers, 2013 | No | Yes | Yes | Yes | Yes | Yes | Yes | Yes | Low |
| Kwan, 2018 | Yes | Yes | Yes | Yes | Yes | Yes | Yes | Yes | Low |
| Pedrana, 2012 | Yes | Yes | Yes | Yes | Yes | Yes | Yes | Yes | Low |
| Bogale, 2010 | No | Yes | Yes | Yes | No | No | Yes | Yes | Moderate |

*Mixed Methods Studies (Mixed Methods Appraisal Tool -* [*http://mixedmethodsappraisaltoolpublic.pbworks.com/w/file/fetch/127916259/MMAT_2018_criteria-manual_2018-08-01_ENG.pdf*](http://mixedmethodsappraisaltoolpublic.pbworks.com/w/file/fetch/127916259/MMAT_2018_criteria-manual_2018-08-01_ENG.pdf)*)*

| Kuhns, 2021 | Ybarra, 2019 | Gannon, 2020 |  |  |
| --- | --- | --- | --- | --- |
| Yes | Yes | Yes | Are there clear research questions? | Screening Questions |
| Yes | Yes | Yes | Do the collected data allow to address the research questions? |  |
| Yes | Yes | Yes | Is the qualitative approach appropriate to answer the research question? | Qualitative |
| Yes | Yes | Yes | Are the qualitative data collection methods adequate to address the research question |  |
| Yes | Yes | Yes | Are the findings adequately derived from the data? |  |
| Yes | Yes | Yes | Is the interpretation of results sufficiently sustained by data? |  |
| Yes | Yes | Yes | Is there coherence between qualitative data sources, collection, analysis and interpretation? |  |
| Not Applicable | Yes | Not Applicable | Is randomisation appropriately performed? | Quantitative RCT |
| Not Applicable | No | Not Applicable | Are the groups comparable at baseline? |  |
| Not Applicable | Yes | Not Applicable | Are there complete outcome data? |  |
| Not Applicable | Unclear | Not Applicable | Are outcome assessors blinded to the intervention provided? |  |
| Not Applicable | No | Not Applicable | Did the participants adhere to the assigned intervention? |  |
| Not Applicable | Not Applicable | Not Applicable | Are the participants representative of the target population? | Quantitative non-randomised |
| Not Applicable | Not Applicable | Not Applicable | Are measurements appropriate regarding both the outcome and intervention (or exposure)? |  |
| Not Applicable | Not Applicable | Not Applicable | Are there complete outcome data? |  |
| Not Applicable | Not Applicable | Not Applicable | Are the confounders accounted for in the design and analysis? |  |
| Not Applicable | Not Applicable | Not Applicable | During the study period, is the intervention administered (or exposure occurred) as intended? |  |
| Yes | Not Applicable | Yes | Is the sampling strategy relevant to address the research question? | Quantitative descriptive |
| Yes | Not Applicable | Yes | Is the sample representative of the target population? |  |
| Yes | Not Applicable | Yes | Are the measurements appropriate? |  |
| Yes | Not Applicable | Yes | Is the risk of nonresponse bias low? |  |
| Not Applicable | Not Applicable | Not Applicable | Is the statistical analysis appropriate to answer the research question? |  |
| Yes | Yes | Yes | Is there an adequate rationale for using a mixed methods design to address the research question? | Mixed Methods |
| Yes | Yes | Yes | Are the different components of the study effectively integrated to answer the research question? |  |
| Yes | Yes | Yes | Are the outputs of the integration of qualitative and quantitative components adequately interpreted? |  |
| Yes | Yes | Yes | Are divergences and inconsistencies between quantitative and qualitative results adequately addressed? |  |
| Yes | Yes | Yes | Do the different components of the study adhere to the quality criteria of each tradition of the methods involved? |  |
| Low | Low | Low | Overall risk of bias | |
