# Supplementary material for: Prevent with Pleasure: A systematic review of HIV public communication campaigns incorporating a pleasure-based approach
Source: PLOS Glob Public Health. 2025 Mar 10;5(3):e0004005. doi: 10.1371/journal.pgph.0004005 (PMC11892838; doi:10.1371/journal.pgph.0004005)
Supplement: S3 Table — (DOCX) [file pgph.0004005.s004.docx]

**Appendix 4 – Study characteristics and details of pleasure operationalisation within included interventions**

|  | | | | | | | | |
| --- | --- | --- | --- | --- | --- | --- | --- | --- |
| **Interventions** | **First Author (Year)** | **Country of Intervention** | **Intervention Modality** | **Study quality** | **Study Design** | **Target Audience of Intervention** | **Aspect of Pleasure Operationalised** | **Operationalisation of Pleasure** |
| *#PrEP4Love* | Phillips (2020) | USA | Website, Advertisements and Pop-Up events | Low | Cross-sectional | Black men who have sex with men, Transgender women of colour, Black cisgender heterosexual women | Enjoyment | The campaign prioritised intimacy and desire, framing consensual sexual activities as healthy and pleasurable. The campaign paired fear-based epidemiological language commonly used to describe HIV infection (e.g., transmit, catch) with words describing positive aspects of sex and intimacy (e.g., desire, love). |
|  | Phillips (2020) |  |  | Moderate | Cross-sectional |  |  |  |
|  | Dehlin (2019) |  |  | Moderate | Cross-sectional |  |  |  |
|  | Keene (2020) |  |  | Moderate | Qualitative |  |  |  |
| *Oral HIV/AIDS Prevention Messaging for illiterate Ethiopian Women* | Bogale (2010) | Ethiopia | Radio messaging | Moderate | Cross-sectional | Illiterate cisgender women | Enjoyment  Empowerment | Discussions between women on the importance of safe sex integrated messages of desire and autonomy, such as ‘*to accomplish your plan and desire […] it is necessary to use a condom’.* |
| *Just/Us* | Bull (2012) | USA | Social media | Moderate | Randomised Controlled Trial | Young people | Empowerment | Content was based around the principle that sexual health is a human right and a function of social justice, consisting of skill-building sessions on topics such as condom negotiation. |
| *Testing Makes Us Stronger* | Habarta (2017) | USA | Magazine adverts, Transit and Billboards | Low | Cross-Sectional | Young Black men who have sex with men | Emotional Connection  Empowerment | Communicated positive, empowering message that HIV testing was a source of strength, communicating a sense of togetherness, community, trust in partners and unitedness. |
|  | Badal (2019) |  |  | Low | Cross-Sectional |  |  |  |
|  | Boudewyns (2018) |  |  | Low | Quasi-experimental |  |  |  |
| *Social Media Intervention Promoting HIV Testing* | Cao (2019) | China | Social media and Messaging platform | Low | Cross-sectional | Men who have sex with men | Emotional Connection | Messages were based around the positive impact of engaging with prevention on affection between partners. Examples include *‘the best love originates from cherishing each other’* and *‘early prevention […] will lead to long-lasting romance and long-lasting life’*. |
| *MyPEEPS Mobile* | Schnall (2018) | USA | Mobile Application | Moderate | Qualitative | Young men who have sex with men | Empowerment | Focuses on building knowledge and skills for safer sex among young men who have sex with men, specifically addressing minority stress, developing risk-reduction plans and promoting condom negotiation. A running theme throughout the intervention is the *‘Bottom Line’* in which participants can set goals about their risk reduction and commit to how much sexual risk they are willing to undertake. |
|  | Ignacio (2019) |  |  | Moderate | Pre-post study |  |  |  |
|  | Gannon (2020) |  |  | Low | Mixed methods |  |  |  |
|  | Cordoba (2021) |  |  | Moderate | Qualitative |  |  |  |
|  | Schnall (2022) |  |  | Low | Randomised Controlled Trial |  |  |  |
| *Make Your Position Clear* | Flowers (2013) | Scotland | Posters, electronic images and leaflets, campaign websites | Low | Cross-sectional | Men who have sex with men | Enjoyment  Empowerment | The campaign was based around the term ‘position’, taken in both a sexual sense (i.e., sexual positionality) and an assertive, empowering sense. Posters were also purposefully hung in venues associated with the pursual of sexual pleasure, such as saunas, gay bars and gay clubs. |
| *Safetxt* | McCarthy (2016) | UK | Text messaging | Moderate | Randomised Controlled Trial | Young People | Empowerment | Messaging provided non-judgemental, non-stigmatising information about STIs, including negotiating condom use and helping others resolve their condom use problems |
|  | Berendes (2023) |  |  | Low | Qualitative |  |  |  |
|  | Free (2022) |  |  | Moderate | Randomised Controlled Trial |  |  |  |
| *Healthempowerment.org* | Hightow-Weidman (2011) | USA | Website | Moderate | Qualitative | Young Black men who have sex with men | Empowerment | The website contained sections providing empowering narratives of safer sex, relationships and sexual orientation, with skill-building sessions centred around using condoms and negotiating safer sex |
| *Love, Sex and Choices* | Jones (2021) | USA | Web-based video series | Low | Quasi-Experimental | Young cisgender Black women in heterosexual relationships | Enjoyment  Empowerment | The story was based around characters becoming more aware of their own worth, making choices intentionally and taking the steps to make those choices happen, promoting sexual autonomy and the ability to attain the choices that participants intend to make. |
|  | Jones (2018) |  |  | Low | Pre-Post study |  |  |  |
|  | Jones (2012) |  |  | Moderate | Randomised Controlled Trial |  |  |  |
| *Guide Enhanced Love, Sex and Choices* | Jones (2015) | USA | Web-based video-series | Low | Quasi-Experimental | Young cisgender Black women in heterosexual relationships | Enjoyment  Empowerment | As above, the story was based around characters becoming more aware of their own worth, making choices intentionally and taking the steps to make those choices happen, promoting sexual autonomy and the ability to attain the choices that participants intend to make. This intervention included a virtual guide to support participants through the intervention. |
| *LifeSkills Mobile* | Kuhns (2021) | USA | Mobile application | Low | Mixed methods | Young transgender women | Empowerment | The intervention addressed the structural and interpersonal challenges of HIV prevention among young transgender women, promoting HIV-related information and motivation to reduce risk and improve behavioural skills. |
| *‘Get an early check – chrysanthemum tea’* | Kwan (2018) | China | Web-based video, posters, leaflets, website, fake ‘chrysanthemum tea’ vending machines | Low | Cross-sectional | Men who have sex with men | Emotional Connection | The video presented a story of two young men who have sex with men in a romantic relationship, promoting the positive outcomes of HIV testing uptake, such increasing dyadic trust. |
| *SMS-Based Interventions on VMMC Uptake* | Leiby (2016) | Zambia | Text messaging | Low | Randomised Controlled Trial | Cisgender men | Enjoyment | Examples of sex-positive messages sent in the campaign include, *‘Surveys in Zambia found that most women who know about MC [male circumcision] prefer circumcised men. Top reason is disease prevention, 2^nd^ reason is sexual satisfaction’*. |
| *Nalamanda’s Radio and Theatre Programme* | Nambiar (2011) | India | Radio | Low | Quasi-Experimental | AIDS Patients | Empowerment | Radio programming compromised short messages relating to the basics of HIV transmission and prevention, with messages addressing women’s health and rights in an HIV context, as well as legal rights when returning home from hospital. |
| *Skyddslaget* | Nielson (2020) | Sweden | Mobile application | Moderate | Qualitative | Young people | Enjoyment  Empowerment | The application provided information on preventive behaviours, specifically condoms, and questioned assumptions, such as that sex with a condom is not good, including a quiz on how to make condoms sexy and a game on discussing condoms with partners in an intimate manner. |
| *Safer Sex Maintenance Text Messages* | Patterson (2020) | Mexico | Text messaging | Low | Randomised Controlled Trial | Female sex workers | Empowerment | The intervention consisted of tailored text messaging, integrating stressors identified by the participant which could represent a potential lapse in safer sex (e.g., *Friend, divorce is stressful but if you stick with your goal of always using condoms with clients, you will feel good about yourself*) |
| *Drama Downunder* | Pedrana (2014) | Australia | Radio, printed resources, outdoor advertisements, public events, banner on gay dating apps | Moderate | Qualitative | Men who have sex with men | Enjoyment | Posters contained headlines such as *‘Get up-front about sexual health! You can get an STI in your arse even if you never get fucked!’* alongside erotic pictures of naked cisgender men. |
|  | Wilkinson (2016) |  |  | Low | Pre-post study |  |  |  |
|  | Pedrana (2012) |  |  | Low | Cross-Sectional |  |  |  |
| *United Against AIDS* | Prati (2016) | Italy | Television, radio, posters, brochures, web-based adverts, cinema/newspaper adverts | Low | Quasi-Experimental | n/a (untargeted) | Emotional Connection  Empowerment | The campaign used messaging aimed at creating a sense of community in HIV response, recognising the collective benefits of safer sex behaviour and HIV testing. |
|  | Prati (2016) |  |  | Low | Quasi-Experimental |  |  |  |
| *Text Me, Girl!* | Reback (2021) | USA | Text messaging | Moderate | Randomised Controlled Trial | Young trans women | Emotional Connection  Enjoyment  Empowerment | Examples of sex-positive messages used in the campaign include, *‘be smart and sexy’, ‘when you stay in HIV care you can expose your heart, not your partner’* and *‘you can take care of yourself and your trans community, take your meds’*. |
| *mHealth-based approach as an HIV prevention strategy among people who use drugs on Pre-exposure Prophylaxis* | Shrestha (2020) | USA | Text messaging | Low | Pre-post study | People who use drugs | Emotional Connection  Enjoyment  Empowerment | Sex-positive messages used in the intervention include, *‘condoms = peace of mind. Wear one and make sure your partner does too’*, *‘great lovers use condoms, always have it handy’* and *‘condoms are sexy. STIs and reinfection are not*’, utilising condom eroticisation and promoting increased trust between partners who use protection. |
| *Trans Women Connected* | Sun (2020) | USA | Mobile application | Moderate | Pre-post study | Transgender women | Emotional Connection  Enjoyment | Part of the intervention promoted pre-exposure-prophylaxis through a sex-positive lens, with the section ‘*Is Pre-exposure prophylaxis right for me or my partners’* including reasons to use pre-exposure prophylaxis, including *‘feeling safer’, ‘feeling more in control’, ‘more intimacy’, ‘taking charge of my sexual health’* and *‘better than condoms’*. |
| *People Like Us* | Tan (2022) | Singapore | Web-based video | Moderate | Randomised Controlled Trial | Men who have sex with men | Empowerment | Messages used in the campaign include those promoting safe-sex negotiation self-efficacy and building confidence practicing safer sex |
| *Crowdsourced HIV Test Promotion Video* | Tang (2016) | China | Web-based video | Low | Randomised Controlled Trial | Men who have sex with men | Emotional Connection | The video describes two men who have sex with men meeting, getting into a relationship and falling in love. The video follows the couple engaging in HIV testing behaviour together, with the final message of the video being, *‘Do regular test for love’*. |
| *ProjectHeartForGirls.com* | Widman (2016) | USA | Website | Moderate | Qualitative | Adolescent cisgender women | Empowerment | The final component of the intervention is an interactive sexual communication skills training exercise to build skills in sexual assertiveness and refusal |
| *Texting 4 Sexual Health* | Yao (2018) | USA | Text messaging | Low | Pre-post study | American Indian and Alaskan Native youth | Enjoyment | Examples of some of the sex-positive messages included in the intervention are, *‘think using a condom will kill the mood? Getting an STD will kill the mood too! It’s easier to enjoy sex when it’s safe :)’*. |
| *InThisTogether* | Ybarra (2021) | Uganda | Text messaging | Moderate | Randomised Controlled Trial | Young people | Empowerment | The final topic of the intervention promotes behavioural skills such as how to negotiate condom use with a partner, integrating content on the societal expectations for gendered sexual interactions between partners and effective communication strategies within the Ugandan context |
| *Guy2Guy* | Ybarra (2018) | USA | Text messaging | Moderate | Randomised Controlled Trial | Young cisgender men who have sex with men | Enjoyment  Emotional Connection | Interventions included practical information on how to increase the physical pleasure associated with using prevention, including using lubrication. Sex-positive intervention messages also included, *‘we have sex for lots of reasons: it feels good, it can be a very personal way of showing your partner you care about them’* and *‘there are lots of good reasons to wait: you can practice […] non-sex things that feel good sexually, like kissing and hand jobs’.* |
|  | Ybarra (2019) |  |  | Low | Mixed methods |  |  |  |
|  | Ybarra (2017) |  |  | Moderate | Randomised Controlled Trial |  |  |  |
| *Ygetit?* | Aladin (2023) | USA | Mobile application and digital comic series | Moderate | Randomised Controlled Trial | Young adults | Enjoyment | Part of the intervention includes a comic strip describing a cisgender woman buying condoms from a cisgender man for a safe sex van, flirting and encouraging the man to come find her at the safe sex van. |
