# Supplementary material for: Prevent with Pleasure: A systematic review of HIV public communication campaigns incorporating a pleasure-based approach
Source: PLOS Glob Public Health. 2025 Mar 10;5(3):e0004005. doi: 10.1371/journal.pgph.0004005 (PMC11892838; doi:10.1371/journal.pgph.0004005)
Supplement: S4 Table — (DOCX) [file pgph.0004005.s005.docx]

**S4 Table – Details of the outcomes reported by included studies that evaluate the efficacy of interventions**

| **Table 4.** Details of the outcomes reported by included studies that evaluate the efficacy of interventions. | | | | | | |  |
| --- | --- | --- | --- | --- | --- | --- | --- |
| **Campaign** | **Study** | **Outcome Coding** | **Type of Outcomes Recorded** | **Follow-up Period** | **Demographic Group** | **Outcome Measure** | **Date Extracted and Author Extracting** |
| *#PrEP4Love* | Phillips, 2020 | Biomedical Prevention | Initiation of pre-exposure prophylaxis | n/a | Young Men who have Sex with Men | Across all districts of Chicago, the proportion of individuals who saw #PrEP4Love adverts and did not initiate use of pre-exposure prophylaxis was between 50-60%. The proportion of participants who saw adverts for pre-exposure prophylaxis and subsequently initiated use ranged from 11.5-30.8% across the regions of Chicago analysed. | Extracted by Luke Muschialli in May 2024 |
|  | Phillips, 2020 | Health Behaviour Change | Having had a conversation with a medical provider about pre-exposure prophylaxis | n/a | Young Men who have Sex with Men | Individuals who had seen the campaign advertisements were significantly more likely to have had a conversation about pre-exposure prophylaxis with a medical provider (odds ratio = 2.52, 95% confidence interval 1.69-3.75). | Extracted by Luke Muschialli in May 2024 |
|  |  | Health Behaviour Change | Being out to a medical provider | n/a | Young Men who have Sex with Men | Individuals who had seen the campaign advertisements were significantly more likely to be out to their medical provider (odds ratio = 1.67, 95% confidence interval = 1.00-2.82). | Extracted by Luke Muschialli in May 2024 |
|  |  | Biomedical Prevention | Uptake of pre-exposure prophylaxis in the past six months | n/a | Young Men who have Sex with Men | Individuals who had seen campaign advertisements were significantly more likely to have taken pre-exposure prophylaxis in the past 6 months (odds ratio = 1.99, 95% confidence interval = 1.20-3.30) | Extracted by Luke Muschialli in May 2024 |
|  |  | Attitudinal Change | Perceived approval from peers on pre-exposure prophylaxis | n/a | Young Men who have Sex with Men | Individuals who had seen the campaign advertisements were significantly more likely to perceive their gay and bisexual male friends as strongly approving of pre-exposure prophylaxis (adjusted odds ratio = 3.85, 95% confidence interval = 1.28-11.60). They were also more likely to believe that all gay and bisexual men in the country strongly approved of pre-exposure prophylaxis (adjusted odds ratio = 8.02, 95% confidence interval = 1.78-36.1). | Extracted by Luke Muschialli in May 2024 |
|  |  | Attitudinal Change | Perception of community uptake of pre-exposure prophylaxis | n/a | Young Men who have Sex with Men | Individuals who had see campaign advertisements were more likely to perceive that almost all of their gay and bisexual male friends were on pre-exposure prophylaxis (adjusted odds ratio = 3.59, 95% confidence interval 1.18-10.90) and were more likely to know someone on pre-exposure prophylaxis (adjusted odds ratio = 2.85, 95% confidence interval = 1.95-4.17). | Extracted by Luke Muschialli in May 2024 |
| *Testing Makes Us Stronger* | Habarta, 2017 | HIV Testing | Self-reported HIV testing | 6 months | Black Men who have Sex with Men | Those who reported exposure to the campaign had 1.38 times the probability of reporting that they got tested for HIV compared to those not reporting exposure (probability ratio = 1.38, 95% confidence interval = 1.11-1.73) | Extracted by Luke Muschialli in May 2024 |
|  |  |  |  | 12 months | Black Men who have Sex with Men | Those who reported exposure to the campaign had 1.48 times the probability of reporting that they got tested for HIV compared to those not reporting exposure (probability ratio = 1.48, 95% confidence interval = 1.07-2.03) | Extracted by Luke Muschialli in May 2024 |
|  | Badal, 2019 | Attitudinal Change | Perceived norms about HIV/AIDS | n/a | Black Men who have Sex with Men | The association between campaign exposure and the following perceived norms were statistically significant: *‘Most people who are important to me think I should get tested for HIV at least every 6 months’* (g statistic = 0.13, *p* = 0.041), *‘Intentions to get tested in next 6 months’* (g = 0.23, *p* = 0.006). The association between campaign exposure and the belief that *‘Most of my sexual partners get tested for HIV at least every 6 months’* (g = 0.12, *p* = 0.053) and *‘Most gay men I know get tested for HIV at least every 6 months’* (g = 0.05, *p* = 0.421) were statistically insignificant. | Extracted by Luke Muschialli in May 2024 |
|  |  | Attitudinal Change | Belief of behavioural messages associated with campaign | n/a | Black Men who have Sex with Men | The association between campaign exposure and the following attitudinal beliefs were statistically significant: ‘*Getting an HIV test is free, fast and confidential*’ (g statistic = 0.33, *p* = < 0.0001), *‘Getting tested for HIV keeps me healthy*’ (g = 0.25, *p* = 0.007), *‘Getting tested for HIV will help me stay strong and informed* (g = 0.28, *p* = 0.003). The association between campaign exposure and the belief that *‘Getting tested for HIV will help me take better care of my sexual partner*’ was statistically insignificant (g = 0.16, *p* = 0.078) | Extracted by Luke Muschialli in May 2024 |
|  |  | Attitudinal Change | Self-efficacy surrounding HIV testing | n/a | Black Men who have Sex with Men | The association between campaign exposure and all measured dimensions of self-efficacy were statistically significant: *‘Could find a free testing site’* (g statistic = 0.33, *p* = 0.000), *‘Could get the results of your HIV test within 20 minutes’* (g = 0.17, *p* = 0.025), *‘Knew that your results would be kept confidential* (g = 0.25, *p* = 0.007). | Extracted by Luke Muschialli in May 2024 |
|  | Boudewyns, 2018 | HIV Testing | Change in number of HIV tests throughout campaign period | n/a | Black Men who have Sex with Men | Before campaign implementation, there was evidence of a month-to-month decrease in HIV testing, decreasing at a rate of 34.96 tests per month (*p* = 0.021), but following introduction of the campaign there was a statistically significant incremental increase in monthly HIV-testing trend by 6.22 testing events per month (*p* = 0.002), indicating a reversal from the pre-campaign downward trend in HIV testing events. This effect was not observed to statistically significant degree in cities in which the campaign was not implemented (*p* = 0.571). | Extracted by Luke Muschialli in May 2024 |
| *Just/Us* | Bull, 2012 | Condom Use | Condom use at last sex | 2 months | Young People | 68% of intervention participants had used a condom at last sex (95% confidence interval = 61-74%) compared to 56% of controls (95% confidence interval = 48-64%) | Extracted by Luke Muschialli in May 2024 |
|  |  |  |  | 6 months | Young People | 60% of intervention participants had used a condom at last sex (95% confidence interval = 52-68%) compared to 61% of controls (95% confidence interval = 52-69%). There was a statistically significant time*condition interaction (F statistic = 3.30, *p* = 0.037) | Extracted by Luke Muschialli in May 2024 |
|  |  | Condom Use | Proportion of protected sex acts | 2 months | Young People | 62% of sex acts were protected amongst intervention participants (95% confidence interval = 57-68%) compared to 57% for controls (95% confidence interval = 51-63%). | Extracted by Luke Muschialli in May 2024 |
|  |  |  |  | 6 months | Young People | 55% of sex acts were protected amongst intervention participants (95% confidence interval = 49-62%) compared to 60% of controls (95% confidence interval = 54-67%). There was a statistically significant time*condition interaction (F statistic = 3.63, *p* = 0.027). | Extracted by Luke Muschialli in May 2024 |
|  |  | Attitudinal Beliefs | Condom self-efficacy (*are you confident using condoms?)* | 2 months | Young people | Measured on a 5-point Likert scale from *all the time* to *never*, mean condom self-efficacy was 3.42 for intervention participants (95% confidence interval = 3.34-3.50) and 3.43 for controls (95% confidence interval = 3.33-3.53). | Extracted by Luke Muschialli in May 2024 |
|  |  |  |  | 6 months | Young people | Mean condom self-efficacy was 3.41 for intervention participants (95% confidence interval = 3.31-3.50) and 3.51 for controls (95% confidence interval = 3.42-3.61). There was no statistically significant time*condition interaction (F statistic = 0.95, *p* = 0.39). | Extracted by Luke Muschialli in May 2024 |
|  |  | Attitudinal Beliefs | Condom norms (are *your friends on Facebook likely to use condoms*) | 2 months | Young people | Measured on a 5-point Likert scale from *all the time* to *never*, mean condom norms for intervention participants was 5.70 (95% confidence interval = 5.49-5.90) and 5.75 (95% confidence interval = 5.52-5.97) for controls. | Extracted by Luke Muschialli in May 2024 |
|  |  |  |  | 6 months | Young people | Mean condom norms for intervention participants was 5.70 (95% confidence interval = 5.47-5.93) and 5.83 for controls (95% confidence interval = 5.60-6.06). There was no statistically significant time*condition interaction (F statistic = 0.69, *p* = 0.50). | Extracted by Luke Muschialli in May 2024 |
|  |  | Attitudinal Beliefs | Proportion of participants intending to use condoms at their next sexual encounter | 2 months | Young people | 87% of intervention participants intended to use a condom at their next sexual encounter (95% confidence interval = 83-91%) compared to 83% of controls (95% confidence interval = 77-88%). | Extracted by Luke Muschialli in May 2024 |
|  |  |  |  | 6 months | Young people | 85% of intervention participants intended to use a condom at their next sexual encounter (95% confidence interval = 79-90%) compared to 82% of controls (95% confidence interval = 75-87%). There was no statistically significant time*condition interaction (F statistic = 1.27, *p* = 0.28). | Extracted by Luke Muschialli in May 2024 |
|  |  | Partnership Behaviour | Proportion of participants with 2 or more sexual partners in the past 2 months | 2 months | Young people | 21% of intervention participants had had 2 or more sexual partners in the past 2 months (95% confidence interval = 16-28%) compared to 12% of controls (95% confidence interval = 8-17%). | Extracted by Luke Muschialli in May 2024 |
|  |  |  |  | 6 months | Young people | 25% of intervention participants had had 2 or more sexual partners in the past 2 months (95% confidence interval = 18-34%) compared to 17% of controls (95% confidence interval = 12-24%). There was no statistically significant time*condition interaction (F statistic = 1.05, *p* = 0.35). | Extracted by Luke Muschialli in May 2024 |
|  |  | Health Behaviour Change | Whether participants were drunk or high during their last sexual experience | 2 months | Young people | Measured on a 5-point Likert scale from *all the time* to *never*, mean ratings of whether intervention participants had been drunk or high during recent sexual encounters was 1.77 (95% confidence interval = 1.70-1.85) compared to 1.81 for controls (95% confidence interval = 1.72-1.89). | Extracted by Luke Muschialli in May 2024 |
|  |  |  |  | 6 months | Young people | Mean ratings of whether intervention participants had been drunk or high during recent sexual encounters was 1.86 (95% confidence interval = 1.77-1.94) compared to 1.85 for controls (95% confidence interval = 1.75-1.94). There was no statistically significant time*condition interaction (F statistic = 0.87, *p* = 0.42). | Extracted by Luke Muschialli in May 2024 |
| *Social Media Intervention Promoting HIV Testing* | Cao, 2019 | HIV Testing | Odds of getting a facility-based HIV test during the intervention period | 3 months | Men who have Sex with Men | After adjusting for age, education, income, marital status, city and facility-based HIV testing, individuals who were able to recall campaign information had 59% greater odds of getting facility-based HIV tested during the intervention period than individuals who were not (adjusted odds ratio = 1.59; 95% confidence interval = 1.13-2.24). Similarly, the adjusted odds of getting facility-based HIV tested during the intervention period increased for every additional image or text individuals recalled (adjusted odds ratio = 1.13, 95% confidence interval = 1.02-1.25). | Extracted by Luke Muschialli in May 2024 |
| *MyPEEPS Mobile* | Ignacio, 2019 | Biomedical Prevention | Qualitative accounts of pre- and post-exposure prophylaxis use | 3 months | Young Men who have Sex with Men | A participant explained learning about pre-exposure prophylaxis for the first time, “I didn’t even know there was a medication to prevent, to lower the risk of HIV and like now that I do, like I always tell people…there is this medication out there, it’s just that people don’t know it. They don’t teach this stuff in health class”. Another young man who had sex with men described specific prevention information he retained: “Knowledge that I did gain from this is about post-exposure prophylaxis. Because I didn’t know that. I didn’t know exactly how it worked. I just like if that you were raped or you went through a dangerous situation…and you want to make sure that there’s the lowest chance of you getting HIV that you talk to a doctor, you get post-exposure prophylaxis within 3 days of the incident and then you go through that, I believe a month, is what the app said”. Another participant also commented on the pre-exposure prophylaxis resources provided, “I thought the activities were pretty easy and straightforward to complete…most of them gave a lot of useful information and at the end, they would give, some were phone numbers or websites were you could locate HIV testing, where you could get PrEP…and I thought that was really useful”. | Extracted by Luke Muschialli in May 2024 |
|  |  | Condom Use | Qualitative accounts of condom use | 3 months | Young Men who have Sex with Men | Another participant described how he believed that the app may change his sexual risk behaviour. He said, “Coming from someone who typically did not use protection when engaged in sexual activities, I started using protection during sexual activities…because of the app. I learned a lot of different consequences, as well as other things you can get from not using protection…I guess I just learned more about myself”. Another participant described adopting safer-sex practices: “Well I can show more restraint now and for example, just last night, I was hanging out with someone that I’m really into and we were close to having sex, but I told him no because we didn’t have a condom, I don’t know his status and I don’t know my own either and I don’t even know if he knows his. I’ve actually been able to share some of the information with another friend of mine” | Extracted by Luke Muschialli in May 2024 |
|  |  | Health Behaviour Change | Qualitative accounts of substance use during sex | 3 months | Young Men who have Sex with Men | A 15-year-old described how he is going to change his risk behaviours and detailed, “I’m definitely not going to do it with people who are drunk or stoned…and also make sure that I definitely have a condom on me or like, make sure that I definitely know…they are tested and make sure I don’t get any HIV or anything”. Another participant described the value of learning about the harmful effects of substance use. He stated, “I learned a lot. I had no knowledge at all of poppers. I think that was really the one that stood out to me…and it’s funny because I had done this activity…learned about them and then a friend of mine mentioned them. It’s just something that I’m so glad I learned about on the app beforehand because had I learned about them from my friend as a source, then I’m sure I would have gotten a very one-sided explanation and description of them. And so, I was really thankful to learn about the dangers”. Another participant described the value of learning the signs of intoxication: “I knew about drugs but didn’t know how to identify when someone is under the influence…or what this or that could potentially do if I really used the wrong way…so I think that was very good”. Another participant described the value of learning about high-risk situations: “We get a lot of that [drug and alcohol information], and I felt it was good, because you don’t think about it in a sexual setting. They don’t really do a lot of relation between drugs and sex…none, actually, in school, so it’s good to actually have that somewhere laid out for you. Because I hadn’t thought about it” | Extracted by Luke Muschialli in May 2024 |
|  |  | Attitudinal Beliefs | HIV Stigma | 3 months | Young Men who have Sex with Men | One participant appreciated the content describing the realities of coping with stigma. He stated, “I personally really enjoyed the ways to manage stigma…it put into words what people, in this community face and like what they actually do to kind of cope with those things… that are like, not necessarily talked about”. Another participant described the immediate lessons learned from the activity: “I think there’s something reassuring about seeing a multitude of ways to manage stigma…it was comforting because I went from having sort of zero concrete ideas…on how to handle stigma to having 4 that I can access at any time if I need”. Lastly, one participant described how the content reflected stressful situations he experienced in high school. He stated, “I related to a lot of these activities in my personal life and I was thinking when I was in a locker room in high school and you know a lot of the guys would mess around, like ‘oh he’s gay,’ not me, they were talking about someone else…like behind the scenes. The gay one. ‘Don’t let him see you.’ You know and I was like bro, ‘[that’s] ignorant’”. | Extracted by Luke Muschialli in May 2024 |
|  | Cordoba, 2021 | Attitudinal Beliefs + HIV Testing + Biomedical Prevention^1^ | Knowledge and awareness of sexual health and HIV | n/a | Young Men who have Sex with Men | Participants expressed that the app increased their awareness of sexual health, including the possibility of beginning pre-exposure prophylaxis, the importance of getting tested for HIV, and practicing HIV-protective behaviours (e.g., condom use): ‘It’s good for constantly reminding you about safe sex practices and definitely keeps you more … something to think about … and makes you more aware of things that maybe you should change about what you do and be more cognizant of your health and other’s health’; ‘It gave me more of a priority to get tested to make sure that I know what’s going on with my body, making sure that I’m taking the necessary steps to stay this way’; ‘Definitely getting tested … obviously … but also trying to stay safe. I do want to be more sexually active, but I do want to be safe, so I’ve been thinking about getting PrEP and also getting tested and making sure that whoever I do it with I have a condom ready and lube because I am uncomfortable a lot of the time, so I have to make sure I get more comfortable.’  Participants felt that the Bottom-Line activity in the app helped them reflect on their own sexual history and behaviours and set boundaries with sexual partner(s): ‘A lot of it was informational for me, just knowing proper condom use, more about STIs, and just safer sex practice. And also, knowing yourself and what you're okay with. So, I feel like the Bottom Line was my personal takeaway that was the #1 for me’. The app also provided decision-making skills and improved sexual health behaviour among some participants: ‘I'm a lot more cautious than I was back then. Because a year ago, I was really reckless. I wouldn’t use a condom because it wasn’t shown a lot in (inaudible) and stuff like that. So, I was having really unsafe sex during that time. But now I'm taking all the precautions’; ‘Conscientious about condom use, I would say. I felt like…requiring myself to wear one. I think that’s probably the biggest takeaway’.  Another skill participants commented on was improved communication skills with partners about safer sex practices: ‘It kind of taught me or it prepared me beforehand how to convince someone to use that (condoms), and how to communicate with your partner, how to do safety precautions without sacrificing the fun’.  After completing each MyPEEPS Mobile activity, participants also felt more confident and knowledgeable about HIV-related topics: ‘There was a lot of information that I wasn’t familiar with before. And I feel glad that I am more familiar with it now so that if a situation did come up, I would be more… I’d feel more confident in handling it’. | Extracted by Luke Muschialli in May 2024 |
|  | Schnall, 2022 | Condom Use | Number of Condomless Sex Acts | 3 months | Young Men who have Sex with men | There was a statistically significant intervention*time incidence rate ratio of 0.56 (95% confidence interval = 0.32-0.99) for intervention participants relative to controls. | Extracted by Luke Muschialli in May 2024 |
|  |  |  |  | 6 months | Young Men who have Sex with Men | There was a non-statistically significant intervention*time incidence rate ratio of 0.61 (95% confidence interval = 0.34-1.08) for intervention participants relative to controls. | Extracted by Luke Muschialli in May 2024 |
|  |  |  |  | 9 months | Young Men who have Sex with Men | There was a non-statistically significant intervention*time incidence rate ratio of 0.83 (95% confidence interval = 0.47-1.47) for intervention participants relative to controls. | Extracted by Luke Muschialli in May 2024 |
|  |  | Partnership Behaviour | Number of sex partners | 3 months | Young Men who have Sex with Men | There was a non-statistically significant intervention*time incidence rate ratio of 1.16 (95% confidence interval = 0.74-1.81) for intervention participants relative to controls. | Extracted by Luke Muschialli in May 2024 |
|  |  |  |  | 6 months | Young Men who have Sex with Men | There was a non-statistically significant intervention*time incidence rate ratio of 0.99 (95% confidence interval = 0.63-1.54) for intervention participants relative to controls. | Extracted by Luke Muschialli in May 2024 |
|  |  |  |  | 9 months | Young Men who have Sex with Men | There was a non-statistically significant intervention*time incidence rate ratio of 1.20 (95% confidence interval = 0.76-1.87) for intervention participants relative to controls. | Extracted by Luke Muschialli in May 2024 |
|  |  | Condom Use | Number of Condomless Anal Sex Partners | 3 months | Young Men who have Sex with Men | There was a non-statistically significant intervention*time incidence rate ratio of 1.09 (95% confidence interval = 0.64-1.87) for intervention participants relative to controls. | Extracted by Luke Muschialli in May 2024 |
|  |  |  |  | 6 months | Young Men who have Sex with Men | There was a non-statistically significant intervention*time incidence rate ratio of 0.73 (95% confidence interval = 0.43-1.26) for intervention participants relative to controls. | Extracted by Luke Muschialli in May 2024 |
|  |  |  |  | 9 months | Young Men who have Sex with Men | There was a non-statistically significant intervention*time incidence rate ratio of 1.27 (95% confidence interval = 0.74-2.17) for intervention participants relative to controls. | Extracted by Luke Muschialli in May 2024 |
|  |  | Health Behaviour Change | Number of sex acts using substances | 3 months | Young Men who have Sex with Men | There was a non-statistically significant intervention*time incidence rate ratio of 0.91 (95% confidence interval = 0.41-1.99) for intervention participants relative to controls. | Extracted by Luke Muschialli in May 2024 |
|  |  |  |  | 6 months | Young Men who have Sex with Men | There was a non-statistically significant intervention*time incidence rate ratio of 0.64 (95% confidence interval = 0.28-1.46) for intervention participants relative to controls. | Extracted by Luke Muschialli in May 2024 |
|  |  |  |  | 9 months | Young Men who have Sex with Men | There was a non-statistically significant intervention*time incidence rate ratio of 1.51 (95% confidence interval = 0.71-3.22) for intervention participants relative to controls. | Extracted by Luke Muschialli in May 2024 |
|  |  | Biomedical Prevention | Use of pre-exposure prophylaxis at follow-up | 3 months | Young Men who have Sex with Men | There was a non-statistically significant intervention*time odds ratio of 1.65 (95% confidence interval = 0.12-23.19) for intervention participants relative to controls. | Extracted by Luke Muschialli in May 2024 |
|  |  |  |  | 6 months | Young Men who have Sex with Men | There was a non-statistically significant intervention*time odds ratio of 5.31 (95% confidence interval = 0.34-83.90) for intervention participants relative to controls. | Extracted by Luke Muschialli in May 2024 |
|  |  |  |  | 9 months | Young Men who have Sex with Men | There was a non-statistically significant intervention*time odds ratio of 3.70 (95% confidence interval = 0.23-58.41) for intervention participants relative to controls. | Extracted by Luke Muschialli in May 2024 |
|  |  | HIV Testing | Engagement with HIV testing at follow-up | 3 months | Young Men who have Sex with Men | There was a non-statistically significant intervention*time odds ratio of 1.18 (95% confidence interval = 0.59-2.35) for intervention participants relative to controls. | Extracted by Luke Muschialli in May 2024 |
|  |  |  |  | 6 months | Young Men who have Sex with Men | There was a non-statistically significant intervention*time odds ratio of 1.28 (95% confidence interval = 0.64-2.60) for intervention participants relative to controls. | Extracted by Luke Muschialli in May 2024 |
|  |  |  |  | 9 months | Young Men who have Sex with Men | There was a non-statistically significant intervention*time odds ratio of 1.72 (95% confidence interval = 0.85-3.47) for intervention participants relative to controls. | Extracted by Luke Muschialli in May 2024 |
| *Makes Your Position Clear* | Flowers, 2013 | HIV Testing | Whether participants have tested for HIV in the previous 6 months | n/a | Men who have Sex with Men | Relative to those reporting no exposure to the campaign, there was a statistically significantly increased odds of having been tested for HIV in the past six months for those reporting high (adjusted Odds Ratio = 1.96, 95% confidence interval = 1.26-3.06) and mid (adjusted Odds Ratio = 1.89, 95% confidence interval 1.24-2.87) exposure to the campaign. There was no statistically significant difference for those reporting low exposure (adjusted odds ratio = 0.90, 95% confidence interval = 0.56-1.42). | Extracted by Luke Muschialli in May 2024 |
|  |  | HIV Testing | Intention to test for HIV in the following 6 months | n/a | Men who have Sex with Men | Relative to those reporting no exposure to the campaign, there was a statistically significantly increased odds of reporting an intention to test for HIV in the following six months for those reporting mid (adjusted odds ratio = 2.01, 95% confidence interval = 1.20-3.36) exposure to the campaign. There was no statistically significant difference for those reporting high exposure (adjusted odds ratio = 1.68, 95% confidence interval = 0.98-2.90). | Extracted by Luke Muschialli in May 2024 |
|  |  | Health Behaviour Change | Use of appropriate lubricant with anal sex in previous year | n/a | Men who have Sex with Men | Relative to those reporting no exposure to the campaign, there was no statistically significant difference in the odds of using appropriate lubricant for anal sex in the past year for those with mid (adjusted odds ratio = 0.71 95% confidence interval = 0.37-1.37) or high (adjusted odds ratio = 0.64, 95% confidence interval = 0.32-1.28) exposure to the campaign. | Extracted by Luke Muschialli in May 2024 |
| *Safetxt* | Free, 2022 | Condom Use | Condom use at last sexual encounter | 4 weeks | Young People | Participants exposed to the intervention had a statistically significantly greater odds of using a condom at their last sexual encounter (odds ratio = 1.12, 95% confidence interval = 1.00-1.25) | Extracted by Luke Muschialli in May 2024 |
|  |  |  |  | 1 year | Young People | Participants exposed to the intervention had a statistically significantly greater odds of using a condom at their last sexual encounter (odds ratio = 1.14, 95% confidence interval = 1.01-1.28) | Extracted by Luke Muschialli in May 2024 |
|  |  | Partnership Behaviour | Reporting less than 2 sexual partners during follow-up | 1 year | Young People | Participants exposed to the intervention had a statistically significantly greater odds of reporting less than 2 sexual partners since joining the trial (odds ratio = 1.11, 95% confidence interval = 1.00-1.24) | Extracted by Luke Muschialli in May 2024 |
|  |  | Partnership Behaviour | Reporting sex with someone new during follow-up | 1 year | Young People | Participants exposed to the intervention had a statistically significantly greater odds of not reporting sex with someone new since joining the trial (odds ratio = 1.13. 95% confidence interval = 1.00-1.28) | Extracted by Luke Muschialli in May 2024 |
|  |  | Condom Use | Condom use at first sex with most recent new partner | 1 year | Young People | Participants exposed to the intervention had a statistically significantly greater odds of reporting condom use at first sex with most recent new partner (odds ratio = 1.27, 95% confidence interval = 1.11-1.45) | Extracted by Luke Muschialli in May 2024 |
|  |  | Attitudinal Change | Self-efficacy for correct condom use | 1 year | Young people | Participants exposed to the intervention reported statistically significantly higher correct condom use self-efficacy (regression coefficient = 0.32, 95% confidence interval = 0.16-0.47). | Extracted by Luke Muschialli in May 2024 |
|  |  | Attitudinal Change | Self-efficacy in negotiating condom use | 1 year | Young People | There was no statistically significant relationship between intervention exposure and reported self-efficacy in negotiating condom use (regression coefficient = 0.03, 95% confidence interval = -0.10-0.17). | Extracted by Luke Muschialli in May 2024 |
| *Love, Sex and Choices* | Jones, 2021 | Attitudinal Change | Response to the question: *Do you think the videos you watched would change a woman’s attitude about having sex when she does not want to?* | n/a | Young Hispanic cisgender women | 45.5% of participants responded *definitely yes*, 33.7% responded *probably*, 18.9% responded *maybe*, 1.9% responded *don’t think so* and 0.0% responded *definitely not*. | Extracted by Luke Muschialli in May 2024 |
|  |  | Attitudinal Change | Response to the question: *Do you think the videos could help a woman decide to leave a man who won’t use condoms?* | n/a | Young Hispanic cisgender women | 9.9% of participants responded *all the time*, 48.5% responded *most of the time*, 15.8% responded *couple of times* and 25.8% responded *don’t know* | Extracted by Luke Muschialli in May 2024 |
|  |  | Attitudinal Change | Response to the question: *Do you think the videos could help a woman learn how to handle herself in a tough situation with a male partner who wants to have unprotected sex when she doesn’t want to?* | n/a | Young Hispanic cisgender women | 18.9% of participants responded *all the time*, 60.4% responded *most of the time*, 11.9% responded *couple of times*, 6.9% responded *don’t know* and 1.9% responded *never*. | Extracted by Luke Muschialli in May 2024 |
|  |  | Attitudinal Change | Response to the question: *Do you think that after watching the videos women will be more likely to get HIV tested?* | n/a | Young Hispanic cisgender women | 32.7% of participants responded *all the time*, 50.5% responded *most of the time*, 5.9% responded *couple of times*, 9.9% responded *don’t know* and 1.0% responded *never*. | Extracted by Luke Muschialli in May 2024 |
|  | Jones, 2018 | Attitudinal Change | Change in high-risk sex script response, assessing the agreement with the premise that condomless sex is needed to begin and preserve a relationship with a man | 6 months | Black cisgender women | Median follow-up high-risk sex script scores were 44.5% as high as the baseline score in the campaign video arm, and 68.0% as high for controls, with a statistically significant greater baseline to follow-up decline in high-risk sex script scores among intervention participants compared to controls (*p* = 0.03). | Extracted by Luke Muschialli in May 2024 |
| *Guide Enhanced Love, Sex and Choices* | Jones, 2015 | Health Behaviour Change | Substance use during sex | 1 month | Black cisgender women | 62.5% of intervention participants compared to 54.5% of controls used alcohol before or during sex in the follow-up period, and 30.0% of intervention participants compared to 20.5% of controls used drugs before or during sex. Both differences were statistically insignificant (*p* = 0.46 and *p* = 0.31, respectively). | Extracted by Luke Muschialli in May 2024 |
|  |  | HIV Testing | Ever had an HIV test | 1 month | Black cisgender women | 77.5% of intervention participants, compared to 75.0% of controls, had ever had an HIV test. This difference was statistically insignificant (*p* = 0.79). | Extracted by Luke Muschialli in May 2024 |
|  |  | HIV Testing | Had an HIV test in the last 3 months | 1 month | Black cisgender women | 45.0% of intervention participants, compared to 36.4% of controls, had had an HIV test in the last 3 months. This difference was statistically insignificant (*p* = 0.43). | Extracted by Luke Muschialli in May 2024 |
|  |  | Partnership Behaviour | Number of sex partners in the previous year | 1 month | Black cisgender women | Mean number of sexual partners for intervention participants was 2.9 (standard deviation = 1.8) compared to 2.6 (standard deviation = 1.8) for controls. This difference was statistically insignificant (*p* = 0.52). | Extracted by Luke Muschialli in May 2024 |
|  |  | Partnership Behaviour | Number of sex partners in the previous three months | 1 month | Black cisgender women | Intervention participants had a mean of 1.6 partners (standard deviation = 0.8) compared to 1.3 (standard deviation = 0.5) for controls. This difference was statistically significant (*p* = 0.03). | Extracted by Luke Muschialli in May 2024 |
|  |  | Condom Use | Had unprotected sex with two or more partners | 1 month | Black cisgender women | 25% of intervention participants, compared to 18.2% of controls, had had unprotected sex with two or more partners during follow up. This difference was statistically insignificant (*p* = 0.45) | Extracted by Luke Muschialli in May 2024 |
|  |  | Condom Use | Had unprotected vaginal sex with a high-risk partner | 1 month | Black cisgender women | 100% of intervention participants, compared to 97.7% of controls, had had unprotected vaginal sex with a high-risk partner during follow up. This difference was not statistically significant (*p* = 0.50). | Extracted by Luke Muschialli in May 2024 |
|  |  | Condom Use | Had unprotected anal sex with a high-risk partner | 1 month | Black cisgender women | 7.5% of intervention participants, compared to 22.7% of controls, had had unprotected anal sex with a high-risk partner during follow up. This difference was not statistically significant (*p* = 0.05). | Extracted by Luke Muschialli in May 2024 |
| *Get an early check – chrysanthemum tea* | Kwan, 2018 | HIV Testing | Uptake of HIV testing in the last 6 months | 6 months | Men who have Sex with Men | Those exposed to the campaign had statistically significantly greater odds of having taken an HIV test in the last 6 months (odds ratio = 2.55, 95% confidence interval = 1.25-5.19) | Extracted by Luke Muschialli in May 2024 |
| *SMS-Based Interventions on VMMC Uptake* | Leiby, 2016 | Health Behaviour Change | Self-reported uptake of voluntary medical male circumcision | 6 months | Cisgender men | There was a statistically insignificant difference in the odds of self-reporting uptake of voluntary medical male circumcision among those received the conventional (odds ratio = 1.13, 95% confidence interval = 0.78-1.65) and the tailored interventions (odds ratio = 1.24, 95% confidence interval = 0.85-1.81) relative to controls. | Extracted by Luke Muschialli in May 2024 |
|  |  | Health Behaviour Change | Verified uptake of voluntary medical male circumcision | 6 months | Cisgender men | There was a statistically insignificant difference in the odds of verified uptake of voluntary medical male circumcision among those received the conventional (odds ratio = 1.21, 95% confidence interval = 0.47-3.09) and the tailored interventions (odds ratio = 0.77, 95% confidence interval = 0.26-2.24) relative to controls. | Extracted by Luke Muschialli in May 2024 |
| *Nalamanda’s Radio and Theatre Programme* | Nambiar, 2011 | Attitudinal Belief | HIV Knowledge | Just exposed (at discharge from ward) | AIDS Patients | Relative to those unexposed, those just exposed to the campaign had a statistically significantly increased HIV knowledge relative to those not exposed (adjusted standardised linear coefficient = 0.16, standard error = 0.04, *p* < 0.05). | Extracted by Luke Muschialli in May 2024 |
|  |  |  |  | 1 month | AIDS Patients | Relative to those unexposed, those exposed a month ago to the campaign had a statistically significantly increased HIV knowledge relative to those not exposed (adjusted standardised linear coefficient = 0.15, standard error = 0.05, *p* < 0.05). | Extracted by Luke Muschialli in May 2024 |
|  |  | Attitudinal Belief | Antiretroviral therapy knowledge | Just exposed (at discharge from ward) | AIDS Patients | Relative to those unexposed, those just exposed to the campaign had a statistically significantly increased antiretroviral therapy knowledge relative to those not exposed (adjusted standardised linear coefficient = 0.20, standard error = 0.03, *p* < 0.01). | Extracted by Luke Muschialli in May 2024 |
|  |  |  |  | 1 month | AIDS Patients | Relative to those unexposed, those exposed a month ago to the campaign had a statistically significantly increased antiretroviral therapy knowledge relative to those not exposed (adjusted standardised linear coefficient = 0.15, standard error = 0.04, *p* < 0.01). | Extracted by Luke Muschialli in May 2024 |
|  |  | Attitudinal Belief | Stigma surrounding people living with HIV | Just exposed (at discharge from ward) | AIDS Patients | Relative to those unexposed, those just exposed to the campaign had statistically significantly reduced disclosure stigma (adjusted standardised linear coefficient = -0.07, standard error = 0.03, *p* < 0.1) and enacted stigma (adjusted standardised linear coefficient = -0.02, standard error = 0.01, *p* < 0.05). There was no statistically significant difference in felt stigma between those just exposed and those not exposed (adjusted standardised linear coefficient = -2.83, standard error = 2.25). | Extracted by Luke Muschialli in May 2024 |
|  |  |  |  | 1 month | AIDS Patients | Relative to those unexposed, those exposed a month ago to the campaign had statistically significantly reduced disclosure stigma (adjusted standardised linear coefficient = -0.07, standard error = 0.04, *p* < 0.1) and enacted stigma (adjusted standardised linear coefficient = -0.02, standard error = 0.01, *p* < 0.01). There was no statistically significant difference in felt stigma between those just exposed and those not exposed (adjusted standardised linear coefficient = -2.23, standard error = 2.68). | Extracted by Luke Muschialli in May 2024 |
|  |  | Condom Use | Used a condom at every sex act | Just exposed (at discharge from ward) | AIDS Patients | Participants just exposed to the campaign had a statistically insignificantly increased odds of reporting using a condom at every sex act compared to those unexposed (adjusted odds ratio = 2.78, standard error = 1.62). | Extracted by Luke Muschialli in May 2024 |
|  |  |  |  | 1 month | AIDS Patients | Participants exposed a month ago to the campaign had a statistically insignificantly increased odds of reporting using a condom at every sex act compared to those unexposed (adjusted odds ratio = 1.28, standard error = 0.84). | Extracted by Luke Muschialli in May 2024 |
|  |  | Health Behaviour Change | Asked the doctor a question about HIV | Just exposed (at discharge from ward) | AIDS Patients | Participants just exposed to the campaign had a statistically significantly increased odds of reporting asking the doctor a question about HIV compared to those unexposed (adjusted odds ratio = 6.32, standard error = 1.95, *p* < 0.05). | Extracted by Luke Muschialli in May 2024 |
|  |  |  |  | 1 month | AIDS Patients | Participants exposed a month ago to the campaign had a statistically insignificant increased odds of reporting asking the doctor a question about HIV compared to those unexposed (adjusted odds ratio = 4.26, standard error = 1.89). | Extracted by Luke Muschialli in May 2024 |
| *Safer Sex Maintenance Text Messages* | Patterson, 2020 | Condom Use | Whether participants had unprotected sex with clients | 24 months | Female sex workers | There was a statistically significant decrease in the reporting of condomless sex with clients among intervention group participants (regression coefficient = 1.61, 95% confidence interval = 0.39-2.83, *p* = 0.01) in the Tijuana region, but this relationship was not statistically significant in the Ciudad Juarez region (regression coefficient = -0.18, 95% confidence interval = -0.62-0.26, *p* = 0.43). | Extracted by Luke Muschialli in May 2024 |
| *Drama Downunder* | Wilkinson, 2016 | HIV Testing | Self-reported HIV testing in the last 12 months | n/a | Men who have Sex with Men | Those who recalled campaign messages correctly had a statistically significantly higher probability of having had an HIV test in the past 12 months relative to those who did not correctly recall campaign messages (incidence rate ratio = 1.30, 95% confidence interval = 1.00-1.70) | Extracted by Luke Muschialli in May 2024 |
|  | Pedrana, 2012 | Attitudinal Beliefs | Sexual Health Knowledge | n/a | Men who have sex with men | Median sexual health knowledge score was not statistically significantly different between participants who were aware and unaware of the campaign (*p* = 0.14). | Extracted by Luke Muschialli in May 2024 |
|  |  | HIV Testing | Clinic-level data on HIV testing | 3 years | Men who have sex with men | There was a statistically significant increase in the average number of HIV tests per month from the pre-campaign period (410 average number of tests per month) to 3 years post-campaign (520 average number of tests per month; *p* < 0.01). | Extracted by Luke Muschialli in May 2024 |
| *United Against AIDS* | Prati, 2016 | Condom Use | Probability of having unprotected sexual intercourse with multiple partners | 6 months | General Population | The probability of having unprotected sexual intercourse with multiple partners decreased significantly among those exposed to the campaign (relative risk = 0.48, *p* = 0.039) and did not change in the unexposed (relative risk = 1.00, *p* = 0.999) | Extracted by Luke Muschialli in May 2024 |
|  |  | Condom Use | Probability of having unprotected sexual intercourse with multiple partners | 6 months | Men who have Sex with Men | The probability of having unprotected sexual intercourse with multiple partners did not decrease significantly among those exposed to the campaign (relative risk = 1.50, *p* = 0.607) or the unexposed (relative risk = 1.33, *p* = 0.999) | Extracted by Luke Muschialli in May 2024 |
|  |  | Condom Use | Probability of having unprotected sexual intercourse with multiple partners | 6 months | Migrants | The probability of having unprotected sexual intercourse with multiple partners did not decrease significantly among those exposed to the campaign (relative risk = 2.00, *p* = 0.508) or the unexposed (relative risk = 1.33, *p* = 0.999) | Extracted by Luke Muschialli in May 2024 |
|  |  | Condom Use | Likelihood of having unprotected sexual intercourse with someone of unknown HIV status | 6 months | General Population | The likelihood of having unprotected sexual intercourse with someone of unknown HIV status did not change significantly in the unexposed (relative risk = 0.40, *p* = 0.453) or the exposed (relative risk = 0.73, *p* = 0.648). | Extracted by Luke Muschialli in May 2024 |
|  |  | Condom Use | Likelihood of having unprotected sexual intercourse with someone of unknown HIV status | 6 months | Men who have Sex with Men | The likelihood of having unprotected sexual intercourse with someone of unknown HIV status decreased significantly in the exposed (relative risk = 11.00, *p* = 0.006) but did not change in the unexposed (relative risk = 0.75, *p* = 0.999). | Extracted by Luke Muschialli in May 2024 |
|  |  | Condom Use | Likelihood of having unprotected sexual intercourse with someone of unknown HIV status | 6 months | Migrants | The likelihood of having unprotected sexual intercourse with someone of unknown HIV status did not change significantly in the unexposed (relative risk = 0.67, *p* = 0.999) or the exposed (relative risk = 5.00, *p* = 0.219). | Extracted by Luke Muschialli in May 2024 |
|  |  | HIV Testing | Lifetime HIV Testing | 6 months | General Population | The probability of undertaking HIV testing did change significantly in the exposed sample (relative risk = 3.04, *p* = <0.001) but not in the unexposed (relative risk = 3.86, *p* = 0.250). | Extracted by Luke Muschialli in May 2024 |
|  |  | HIV Testing | Lifetime HIV Testing | 6 months | Men who have Sex with Men | The probability of undertaking HIV testing did not change significantly in the exposed (relative risk = 4.63, *p* = 0.063) or the unexposed (relative risk = 1.00, *p* = 0.999) | Extracted by Luke Muschialli in May 2024 |
|  |  | HIV Testing | Lifetime HIV Testing | 6 months | Migrants | No migrant participants had undertaken HIV testing after the campaign | Extracted by Luke Muschialli in May 2024 |
|  | Prati, 2016 | Health Behaviour Change | Number of calls to the Italian National AIDS Help-Line | 6 months | General population | The daily number of calls at the Italian National AIDS Help-Line significantly increased during the campaign (parameter estimate = 0.478, t = 4.635, *p* < 0.001). | Extracted by Luke Muschialli in May 2024 |
| *Text Me, Girl!* | Reback, 2021 | Biomedical Prevention | Currently on antiretroviral therapy | 18 months | Transgender women | Participants reporting greater intervention exposure had a statistically insignificant difference in the odds of currently being on antiretroviral therapy (adjusted odds ratio = 1.24, 95% confidence interval = 0.99-1.54) | Extracted by Luke Muschialli in May 2024 |
|  |  | Health Behaviour Change | Attended an HIV care visit in past 6 months | 18 months | Transgender women | Participants reporting greater intervention exposure had a statistically significantly increased odds of having attended an HIV care visit in the past 6 months (adjusted odds ratio = 1.33, 95% confidence interval = 1.10-1.61). | Extracted by Luke Muschialli in May 2024 |
|  |  | Biomedical Prevention | Antiretroviral therapy adherence | 18 months | Transgender women | Participants reporting greater intervention exposure had a statistically insignificant difference in the odds of adhering to antiretroviral therapy therapy (adjusted odds ratio = 1.01, 95% confidence interval = 0.84-1.20) | Extracted by Luke Muschialli in May 2024 |
| *Trans Women Connected* | Sun, 2020 | Attitudinal Beliefs | Knowledge of pre-exposure prophylaxis and intentions | n/a | Transgender women | Following intervention, participants reported a statistically significantly greater knowledge of pre-exposure prophylaxis (*p* = 0.008). There was no statistically significant change in intention to seek more information about pre-exposure prophylaxis (*p* = 0.58), intention to discuss pre-exposure prophylaxis with providers (*p* > 0.99), intention to discuss pre-exposure prophylaxis with a partner (*p* > 0.99) or self-efficacy in discussing pre-exposure prophylaxis (*p* = 0.16). | Extracted by Luke Muschialli in May 2024 |
| *People Like Us* | Tan, 2022 | HIV Testing | Ever tested for HIV | 6 months | Men who have Sex with Men | There was no statistically significant relationship between intervention exposure and having ever tested for HIV (*p* = 0.85) | Extracted by Luke Muschialli in May 2024 |
|  |  | HIV Testing | Tested for HIV in the last 6 months | 6 months | Men who have Sex with Men | There was no statistically significant relationship between intervention exposure and having tested for HIV in the past 6 months (*p* = 0.41) | Extracted by Luke Muschialli in May 2024 |
|  |  | HIV Testing | Tested regularly (at least yearly) for HIV | 6 months | Men who have Sex with Men | Intervention participants were significantly more likely to report testing regularly for HIV than controls (*p* = 0.02) | Extracted by Luke Muschialli in May 2024 |
|  |  | HIV Testing | Intention to test for HIV in the next 3 months | 6 months | Men who have Sex with Men | Intervention participants were statistically more likely to report an intention to test for HIV in the next 3 months compared to controls (*p* = 0.009). | Extracted by Luke Muschialli in May 2024 |
|  |  | Attitudinal Beliefs | Perceived HIV risk | 6 months | Men who have Sex with Men | There was no significant difference in reported perceived HIV risk between intervention and control participants (*p* = 0.32) | Extracted by Luke Muschialli in May 2024 |
|  |  | Attitudinal Beliefs | Knowledge of pre- and post-exposure prophylaxis | 6 months | Men who have Sex with Men | There was no significant difference in reported knowledge of HIV pre- (*p* = 0.49) or post-exposure prophylaxis (*p* = 0.43) between intervention and control participants | Extracted by Luke Muschialli in May 2024 |
|  |  | Condom Use | Inconsistent condom use in last 6-months with casual partners | 6 months | Men who have Sex with Men | There was no significant difference in the inconsistent use of condoms in the last 6-months with casual partners between intervention and control participants (*p* = 0.60) | Extracted by Luke Muschialli in May 2024 |
|  |  | Attitudinal Beliefs | HIV self-testing efficacy and HIV testing social norms | 6 months | Men who have Sex with Men | There was no significant difference in HIV self-testing efficacy (*p* = 0.76) or HIV testing social norms (*p* = 0.28) between intervention and control participants. | Extracted by Luke Muschialli in May 2024 |
| *Crowdsourced HIV Test Promotion Video* | Tang, 2016 | Condom Use | Self-reporting of condomless anal sex | 6 months | Men who have Sex with Men | There was no significant difference in the reporting of condomless anal sex between intervention and control participants (*p* = 0.77) | Extracted by Luke Muschialli in May 2024 |
| *Texting 4 Sexual Health* | Yao, 2018 | Attitudinal Belief | Condom use knowledge, attitude and intention | 3 months | Young people | There was no statistically significant change in the odds of reporting increased condom use knowledge (odds ratio = 1.15, 95% confidence interval = 0.57-2.31), intention to ask partners to use condoms (odds ratio = 1.33, 95% confidence interval = 0.77-2.29) or intention to use a condom (odds ratio = 0.97, 95% confidence interval = 0.50-1.89) at follow up. There was a statistically significant increased odds of reporting positive attitudes towards condom use at follow-up (odds ratio = 3.25, 95% confidence interval = 1.44-7.35). |  |
|  |  |  |  | 6 months | Young people | There was no statistically significant change in the odds of reporting increased condom use knowledge (odds ratio = 1.30, 95% confidence interval = 0.62-2.71), intention to ask partners to use condoms (odds ratio = 1.07, 95% confidence interval = 0.61-1.86) or intention to use a condom (odds ratio = 0.84, 95% confidence interval = 0.43-1.66) at follow up. There was a statistically significant increased odds of reporting positive attitudes towards condom use at follow up (odds ratio = 3.93, 95% confidence interval = 1.66-9.34). | Extracted by Luke Muschialli in May 2024 |
|  |  | Condom Use | Always used a condom during follow-up | 3 months | Young people | There was a statistically significant increased odds of participants reporting always using a condom with partners post-intervention (odds ratio = 2.43, 95% confidence interval = 1.15-5.13) | Extracted by Luke Muschialli in May 2024 |
|  |  |  |  | 6 months | Young people | There was a statistically significant increased odds of participants reporting always using a condom with partners post-intervention (odds ratio = 2.60, 95% confidence interval = 1.18-5.73) | Extracted by Luke Muschialli in May 2024 |
| *InThisTogether* | Ybarra, 2021 | Condom Use | Ever condom use in the past 90 days | 5 months | Young people | Intervention participants had a statistically insignificant difference in the odds of ever having used a condom in the past 90 days compared with controls (adjusted odds ratio = 1.38, 95% confidence interval = 0.67-2.83) | Extracted by Luke Muschialli in May 2024 |
|  |  | Condom Use | Number of condom sex acts in the past 90 days | 5 months | Young people | Intervention participants had a statistically significantly higher rates of sex acts with a condom in the past 90 days compared to controls (adjusted incidence rate ratio = 1.82, 95% confidence interval = 1.44-2.28) | Extracted by Luke Muschialli in May 2024 |
|  |  | Health Behaviour Change | Abstinence | 5 months | Young people | Intervention participants had a statistically insignificant difference in the odds of having been abstinent in follow-up compared with controls (adjusted odds ratio = 1.08, 95% confidence interval = 0.47-2.44) | Extracted by Luke Muschialli in May 2024 |
|  |  | HIV Testing | Past 90-day HIV testing | 5 months | Young people | Intervention participants had a statistically significantly higher odds of testing for HIV in the past 90 days compared to controls (adjusted odds ratio = 2.41, 95% confidence interval = 1.11-5.24) | Extracted by Luke Muschialli in May 2024 |
| *Guy2Guy* | Ybarra, 2018 | Attitudinal Beliefs | Membership in a *High Motivation* Latent Class, defined by positive attitudes towards abstinence and condom use | 3 months | Young Men who have Sex with Men | Intervention participants had a statistically significantly higher odds of belonging to the *High Motivation* class than controls (adjusted odds ratio = 2.56, *p* = 0.05), but a statistically insignificant difference in the odds of belonging to the *high condom motivation class* (adjusted odds ratio = 1.12, *p* = 0.85), defined by positive views towards condom use but negative views towards abstinence | Extracted by Luke Muschialli in May 2024 |
|  | Ybarra, 2017 | Condom Use | Number of Condomless Sex Acts | 3 months | Young Men who have Sex with Men | There was a statistically insignificant difference in the incidence of number of condomless sex acts among intervention participants compared to controls (adjusted incidence rate ratio = 1.02, 95% confidence interval = 0.51-2.04) | Extracted by Luke Muschialli in May 2024 |
|  |  | Health Behaviour Change | Abstinence | 3 months | Young Men who have Sex with Men | There was a statistically insignificant difference in the odds of intervention participants reporting abstinence compared to controls (adjusted odds ratio = 0.63, 95% confidence interval = 0.36-1.12), but this effect was statistically significant when investigating abstinence among sexually experienced participants, with intervention participants being significantly less likely to report abstinence at follow-up (adjusted odds ratio = 0.48, 95% confidence interval = 0.23-0.99) | Extracted by Luke Muschialli in May 2024 |
|  |  | HIV Testing | Tested for HIV in follow-up | 3 months | Young Men who have Sex with Men | Intervention participants had a statistically significantly increased odds of having tested for HIV during follow-up compared to controls (adjusted odds ratio = 3.42, 95% confidence interval = 1.65-7.09). | Extracted by Luke Muschialli in May 2024 |

**Notes**: 1. Qualitative quotes captured multiple aspects of HIV prevention thus these results will be described in all relevant categories in the narrative synthesis
